# Estimating the medical capacity required to administer mass prophylaxis: a hypothetical outbreak of smallpox virus infection in Korea

**DOI:** 10.4178/epih.e2019044

**Published:** 2019-10-10

**Authors:** Sangwoo Tak, Soomin Lim, Heesu Kim

**Affiliations:** 1 Institute of Health and Environment, Seoul National University, Seoul, Korea; 2 Department of International Health, Bloomberg School of Public Health, Johns Hopkins University, Baltimore, MD, USA

**Keywords:** Smallpox, Surge capacity, Government, Vaccination

## Abstract

**OBJECTIVES:**

The aim of this study was to estimate the medical surge capacity required for mass prophylaxis based on a hypothetical outbreak of smallpox.

**METHODS:**

We performed a simulation using the Bioterrorism and Epidemic Outbreak Response Model and varied some important parameters, such as the number of core medical personnel and the number of dispensing clinics.

**RESULTS:**

Gaps were identified in the medical surge capacity of the Korean government, especially in the number of medical personnel who could respond to the need for mass prophylaxis against smallpox.

**CONCLUSIONS:**

The Korean government will need to train 1,000 or more medical personnel for such an event, and will need to prepare many more dispensing centers than are currently available.

## INTRODUCTION

Smallpox used to be a major scourge of humankind, with high infection and mortality rates. History shows that 1 smallpox-contagious individual can infect, on average, 3.5-7.0 people naive to the virus [1]. This high infectiousness prompted communities to require at least 70% vaccination coverage for effective herd immunity. As almost one-third of infected people died of severe hemorrhage and dehydration, smallpox posed a uniquely severe threat to public health until its eradication (World Health Organization [WHO], 2014). As a result of an intensified global eradication program led by the WHO from 1967 to 1980, smallpox was declared to be eradicated [2]. Since then, due to the cessation of immunization programs around the world, the majority of the world’s population has now become vulnerable to this virus. Smallpox may pose a threat to public health since the possibility of an intentional or accidental release of the smallpox virus still exists [3]. Currently, vaccination is considered the most effective way to stop a smallpox outbreak [4]. Prior to its eradication, there were 2 strategies of smallpox vaccination: ring vaccination and mass vaccination during and after the incident. Ring vaccination was performed to prevent further exposure to the virus among those who were at risk of exposure and ranged from 1 to 70,000 vaccinations per case [5]. It was usually performed in household members of those who were symptomatic or those who had been exposed to the virus. Mass vaccination is a strategy to vaccinate all eligible people who have no immunity and hence are vulnerable to the disease. Although there is no one-size-fits-all plan for a smallpox outbreak with respect to vaccination, it has been demonstrated that achieving high vaccination coverage was key to eradicating the virus during outbreaks in the past [4]. Therefore, if for any reason smallpox reemerges, regardless of the nature of the reemergence (either intentional or accidental release), mass vaccination may be the best option to attain high immunization coverage among the population, preventing further spread of the disease. Mass prophylaxis covering tens of millions of people must be completed within a short time. Given the dire urgency, once a smallpox outbreak occurs, the plan to distribute and dispense related medical supplies should be implemented effectively. Without properly trained medical personnel and supporting facilities, a successful response to a smallpox outbreak is not guaranteed.

Stockpiled smallpox vaccines were available in only a few countries before 2001, when the 2001 anthrax attack occurred in the USA. Additional production of the vaccinations for smallpox and other diseases was begun to meet any major demand for vaccines in the case of an intentional release of smallpox virus [6]. A few countries have stockpiled smallpox vaccine sufficient to cover 100% of their population [7]. The Korea is a country where an intentional release might occur, as a few publications have pointed out [8]. In response, the Korean government has been working to stockpile smallpox vaccines for at least 80% of the population. The most recent official announcement indicated that the stockpile of smallpox vaccines, including first-generation and second-generation vaccines, would cover 33% of the Korean population [9].

Even if sufficient smallpox vaccines are available when an outbreak occurs, however, it is still necessary to have medical personnel available who can administer prophylactic measures nationwide in an organized manner. Because of the significance of the disease, clinicians may volunteer to help respond to a medical surge during an outbreak. As it is known that at least a week is needed to acquire protection against the virus after vaccination [10], an important question arises: how quickly can millions of people who need the vaccination be effectively protected against the virus while utilizing volunteer clinicians during the outbreak? To meet the demand for mass vaccination, medical personnel should be trained ahead of time. By conducting a national training program for emergency responders to biological events, including bioterrorism attacks, the Korean government has been diligent in promoting the vaccination of medical personnel and emergency responders against smallpox and other potential bioterrorism agents. However, only a small fraction of medical personnel is currently vaccinated and has completed the training to handle a medical surge during a smallpox outbreak.

Like most developed countries in Europe and North America, the Korea is implementing a plan to enhance its capacity to respond to a potential outbreak of smallpox. However, there is a lack of information on the estimated need for medical staff to provide prophylaxis, which is likely to be the most effective intervention in the case of a large smallpox outbreak in Korea. Preparation for a medical surge during a potential outbreak should include a quantitative assessment of the medical resources available for large-scale vaccination. Hence, we aimed to generate quantitative estimates of the response capacity needed for a medical surge during and after a hypothetical smallpox outbreak in the Korea. Specifically, this study focused on the number of core medical staff and dispensing clinics required and the duration needed for successful mass prophylaxis strategies.

## MATERIALS AND METHODS

We used the Weill/Cornell Bioterrorism and Epidemic Outbreak Response Model (BERM version 2.0) to estimate the medical surge. BERM is a mass prophylaxis planning tool that was developed by researchers in the Department of Public Health at Weill Medical College of Cornell University. It calculates the estimated numbers of staff and clinic sites needed to respond to a major disease outbreak or bioterrorism attack on a given population [11]. We repeated these estimations while varying the number of days of the vaccination campaign and the number of medical staff members and dispensing clinics available, as well as some predetermined parameters, such as campaign and clinic characteristics. Campaign characteristics included the size of the population undergoing mass prophylaxis and the duration of the campaign. In this study, we assumed that 35 million people would need vaccination (constituting 70% of the 50 million residents of Korea). This 70% figure represents the lowest amount of coverage required for herd immunity; as such, 35 million served as the target population for prophylaxis required for immediate intervention to control a smallpox outbreak [12]. The duration of the campaign is defined as the time frame taken to complete the prophylaxis campaign. The campaign duration was adjusted within a range of 10 days to 1,080 days. Clinic characteristics included hours of clinic operation, shifts per day, downtime, per-clinic flow rate, crisis/isolation counseling, and patient testing. Hours of clinic operation refers to the hours of operation for the campaign’s dispensing clinics. Shifts per day are the number of anticipated work shifts per day. Downtime is defined as the percentage of time used for breaks or lunch for the average staff worker. We assumed that dispensing clinics would operate for 24 hours with 3 work shifts per day, and downtime was assumed to be 15%, as recommended. Per-clinic flow rate is defined as the rate at which patients enter each dispensing clinic and is calculated as patients/min/clinic. It is the combined outcome of the characteristics related to the workflow planned for dispensing clinics, and it is dependent on several variables, such as the number of briefing rooms available for use, each room’s capacity, and the length of each vaccination session. No research in Korea has examined patient flow or health facilities’ capacity to handle mass vaccination in the context of the Korean healthcare environment. Hence, we used the values recommended by the US Centers for Disease Control and Prevention (CDC) for those characteristics; CDC recommended an average patient flow rate of 10 patients per minute for each dispensing clinic based on the number of briefing rooms available for use (3-5 rooms), the capacity of each room (50-75 patients), and the length of the briefing (20 minutes). We varied the rates a priori from 7.5 to 18.5 patients/min/clinic to examine the resulting changes in the number of medical personnel and the duration of the campaign. In this study, crisis/isolation counseling intended to provide individuals with on-site assistance and referral information was considered. Our simulation did not include the time taken and procedures involved with patient testing to evaluate potential contraindications to the vaccine. The event characteristics included process time scenario, event scenario, and biological agent. The process time scenario refers to the amount of time required to process an individual at each dispensing clinic station. We specified that the biological agent was communicable, chose a fast process time, and chose a large-scale event scenario to reflect the surging demand during a hypothetical outbreak of smallpox. The core staff is defined as those individuals trained to work in direct contact with patients, including triage and medical evaluation staff, form distributors, collectors, and vaccinators. The number of core staff available in our simulation ranged from 50 to 1,200 staff members. The minimum number of core staff was based on the number of medical personnel who have been vaccinated among health professionals in Korea according to the most recent available information. The maximum number of core staff is indicative of the number of medical personnel who should be vaccinated before responding to a smallpox outbreak. We chose 1.200 as the largest input for core medical staff after a series of iterations because it allowed a reasonable range for patient flow given various other inputs.

To find the optimal range for the number of core staff and dispensing clinics and the duration of the campaign, we first computed the total size of the population to be vaccinated given various campaign lengths and numbers of core staff. The results were calculated using the following formula:

(1) $$R_{CAMPAIGN}=Pop÷T=average\;campaign\;flow\;rate\;\left(\frac{patients}{min}\right)$$

WherePop=total size of populationandT=length of time for the campaign

We then calculated the number of dispensing clinics needed given some selected campaign durations and per-clinic patient flow rates. The number of dispensing clinics (N_DVC_) was calculated by dividing the average campaign flow rate by the average clinic patient flow, as follows:

(2) $$N_{DVC}=R_{CAMPAIGN}÷R_{DVC}$$

$$\mathrm{Where}\;R_{CAMPAIGN}=average\;campaign\;flow\;rate\;(patients/min)\;\mathrm{and}\;R_{DVC}=average\;clinic\;flow$$

More detailed information on the parameters and equations used in this simulation can be found from the manual published alongside the calculation worksheets in the analysis tool (BERM version 2.0) [11].

We varied the number of days for the mass vaccination campaign from 10 to 1,082 to examine how many days are feasible for the country to plan on. Burke et al. [13] showed that a smallpox outbreak may continue for up to 1,200 days without any intervention. Other research applied 365 days as a target duration to stop the spread and control smallpox using mass vaccination and quarantine [5]. This iteration was conducted under the assumption that the country will have a sufficient number of dispensing clinics, implemented by letting the parameter float in the model. Also varied was the number of dispensing clinics, to determine the number of clinics that would be reasonable to prepare for an effective campaign. A few requirements for dispensing clinics, such as 24 hours of operation, 3 work shifts per day, and 15% downtime, were considered in the model. Some requirements were not included, such as the existence of buildings for quarantine and isolation that were separate from those for patient testing. Other parameters and variables were determined to reflect the current capacity of Korea and potential milestones for future goals. Table 1 shows the variables that were considered and predetermined prior to our estimation.

### Ethics statement

Ethical approval was not required because this study was based on a series of computer simulations and did not use any human or animal data in the study.

## RESULTS

We estimated the total population that could be vaccinated given the duration of the campaign in days and the number of core medical personnel available, assuming that the number of dispensing clinics was optimal. We iterated this estimation to produce a total of 168 observations with 7 different campaign lengths (10, 30, 90, 180, 360, 720, and 1,080 days) and 24 different numbers of core medical personnel, varying from 50 to 1,200.

**Figure 1.**
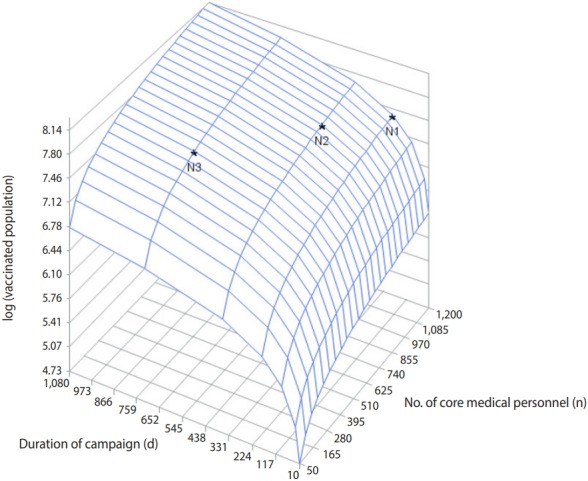
Distribution of the size of the vaccinated population based on various campaign durations and numbers of core medical personnel (N1: core medical personnel=1,200, duration of campaign=180 days; N2: core medical personnel=903, duration of campaign=360 days; N3: core medical personnel=452, duration of campaign=720 days).

Figure 1 shows the total size of the vaccinated population by campaign duration and the number of core staff members available at a flow rate of 10 patients/min/clinic. The total size of the vaccinated population was converted into a log scale: log (vaccinated population). To vaccinate the target population (35 million people) in 180 days after the outbreak, at least 1,200 core medical personnel would be needed to complete prophylaxis (Figure 1, N1). If the vaccination could be performed within 360 days, at least 903 core staff would be needed. With only 452 core medical personnel, it would take at least 720 days to attain the target vaccination goal (Figure 1, N3).

**Figure 2.**
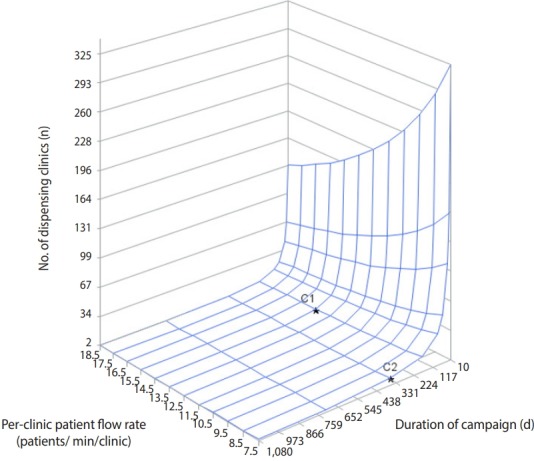
Number of dispensing clinics needed according to campaign duration and per-clinic patient flow rate (C1: per-clinic patient flow rate=14.5, duration of campaign=180 days; C2: per-clinic patient flow rate=7.5, duration of campaign=360 days).

Figure 2 shows the number of dispensing clinics required for vaccination based on patient flow rate per clinic for a given campaign duration. Assuming that sufficient core medical personnel would be available to fully staff the given number of dispensing clinics, the campaign duration and flow rate were inversely correlated with the number of dispensing clinics. At a flow rate of at least 14.5 patients/min/clinic, fewer than 10 clinics would be required to achieve 70% vaccination within 180 days. At a flow rate of 7.5 patients/min/clinic, approximately 10 clinics would be sufficient to achieve 70% vaccination within 365 days of the start of the campaign.

We also examined the effect of patient flow rate on the number of dispensing clinics and core staff per clinic when the campaign duration was fixed at 360 days with approximately 1,000 core medical staff available (Table 2). Because we could not fix the number of core medical staff at exactly 1,000, the results are based on the closest possible estimate of medical staff to 1,000. Table 2 shows the number of dispensing clinics and per-clinic core staff required to meet the per-clinic patient flow rate, assuming that the duration of the campaign would be 360 days. Flow rate was found to be correlated with the number of core staff available. As the flow rate increased, the number of dispensing clinics decreased, while the number of core staff per clinic increased. At a flow rate of 8.5 patients/min/clinic, the number of dispensing clinics required for the campaign was 8, and the number of core staff per clinic was 114, estimated using a total of 912 core staff.

## DISCUSSION

Examining the potential surge of demand for medical resources during a hypothetical smallpox outbreak provides an important opportunity for decision-makers to re-evaluate Korea’s current capacity to meet this demand. From our crude but conservative simulation using a well-established tool, we found that there is expected to be a gap in medical surge capacity if, for any reason, an immediate response to a smallpox outbreak is needed. Even among competing priorities, developing personnel capacity to administer mass prophylaxis for a communicable disease appears to be a top priority.

During a medical surge, the availability of medical staff who are trained and prepared for responding to such an event is of utmost importance for successful and timely response and recovery. During an outbreak, when medical staff members are not properly protected, infected medical personnel can be more than just a burden and could actually increase the risk to the public. The USA has implemented a program to vaccinate medical personnel and emergency responders to enhance preparedness for a smallpox outbreak [1]. While the program addressed many relevant issues, the smallpox vaccination campaign also suffered from low participation. Participants later reported that concerns over possible side-effects of the vaccination kept them from getting vaccinated [14]. Although smallpox vaccination was administered for many decades, it was not always safe to administer due to possible side-effects and medical complications, especially among the immunocompromised [15,16]. This aspect of smallpox vaccination adds complexity to its planning because it requires a much more sophisticated arrangement of related logistics, such as transport, evacuation, and medical facilities in which to dispense the vaccines and quarantine patients. The Korea may benefit from the lessons learned from the experience of the USA in meeting many preparedness demands for a possible biological event, such as one involving the emergence of infectious disease. After the last report of smallpox infection in 1961, the Korea ceased mass vaccination in 1978. Anyone who was born before 1966 fully underwent smallpox vaccination, while anyone who was born after 1978 has no vaccination history for smallpox and is therefore vulnerable to smallpox infection [17]. Immunization with vaccinia-based vaccines involves inoculation of the skin using a bifurcated needle that holds a dose (a small drop) of the vaccine in its fork. This needle is first used to release the liquid onto the skin and is then held perpendicular to the skin; the vaccinator then rapidly and vigorously punctures the skin in an area of about 5 mm in diameter, making a trace of blood appear [12]. This practice requires a skill that is not likely to be taught in most Korean medical schools and hence needs to be learned through hands-on training. Even when a member of the medical personnel (a vaccinator) becomes available after being immunized, her or she must be trained to use the bifurcated needle in this manner before being able to vaccinate patients. This again highlights the importance of advance training of proper medical personnel in preparation for such an event.

As part of the national vaccination plan during an emergency, health centers (and possibly some health posts) across Korea will be used for dispensing vaccines. This availability of health centers implies that proper training of medical staff should be the most urgent task to accomplish. In Korea, doctors are predominantly located in metropolitan areas, leaving remote parts of the country vulnerable to a delayed response if a large-scale infectious disease outbreak occurs. Nurses are less concentrated, but they are still largely located in metropolitan areas. There are about 250 health centers across the country, all of which are run by 1 or more physicians depending on the catchment area [18]. Mass vaccination using these health centers should be part of the national campaign plan of the Korea because it will be much easier to mobilize these facilities and their associated personnel to respond to an event than to do the same for private medical entities. Other medical facilities with certain equipment, such as a negatively pressurized room for highly pathogenic patients, could be used for mass prophylaxis during a medical surge. Such hospitals should have sufficient space for quarantine and isolation facilities, and these should be ready for use. To ensure that prophylactic medications and vaccines reach those affected, each dispensing clinic should be equipped to serve several core functions, such as mass triage, medical evaluation of symptomatic individuals, pharmacotherapeutic consultations for drug or dosage adjustment if needed, and provision of antibiotics or vaccinations [19]. Depending on the clinic’s capacity, it may also perform health emergency surveillance, patient briefings and communication, and mental health counseling. Facilities for shelters and medical evacuation should be also designated for a large-scale smallpox outbreak. A possible option is to use some of the government training facilities across the country that could accommodate large crowds as well as those in need of isolated rooms for quarantine.

Despite the lessons we derived from this simulation, there are limitations in interpreting the results. BERM version 2.0 is a well-designed tool to help prepare for dispensing medical countermeasures during public health emergencies. However, since this tool was designed to apply to the American healthcare setting, parameters used in the tool should not be assumed to apply similarly to the healthcare settings of other countries. In particular, core staff in BERM version 2.0 were defined as those who interact with patients, such as greeters (screeners), form distributors, medical evaluators, briefing supervisors, triage staff, testing staff, vaccinators, crisis counselors, exit educators, and transport providers (emergency medical services). These clinical roles may be filled differently in the Korean healthcare setting. Depending on the availability of trained staff, there may be a discrepancy in the simulation inputs in this regard. However, assignment of such core staff is based on the needs for services provided at the clinic during the mass vaccination in order to assure that there is no backup of patients at the clinic. The inputs for core staff in this simulation study adhered to CDC’s recommendation that over 60% of core staff be assigned as medical evaluators, triage staff, and vaccinators, a distribution pattern that would be crucial during a public health emergency [19]. Additionally, not all fixed parameters used were validated by previous studies. For example, we assumed that the dispensing clinics would operate 24 hours a day with a 15% downtime. Although this value is within the CDC’s recommended range, it has never been field-tested. In reality, the emergency operation of facilities may encounter interruptions due to unforeseen mishaps which may result in longer downtime, implying a need for more medical surge capacity than we estimated.

Our simulation assumed that each dispensing clinic would have the capacity to handle the same number of patients. This allowed us to treat the number of dispensing clinics as a function of the number of core medical staff. As such, a dispensing clinic with 200 core staff members can be considered equivalent to 2 small clinics with 100 core staff members each. We also assumed that the government would have facilities designated ahead of time for mass vaccination with minimum requirements for the point at which these vaccines are dispensed, such as security, crowd control measures, quarantine and isolation rooms, and so on. Adding these complexities to medical surge capacity may require more sophisticated simulations, including, for example, a gradual increase of participating dispensing clinics over time and variation in the patient flow rate between clinics. Several other factors were not included in our simulation: (1) transportation from communities to dispensing clinics, (2) the administrative burden of crowd control near the clinics, and (3) risk communication during the outbreak and promotion of vaccination afterward. The requirements for health facilities administering mass prophylaxis are comprehensive and were well documented in a previous report [19]. However, those guidelines were based on the USA healthcare environment, so caution should be paid when applying them to the healthcare system in Korea. The distribution of patients across the country may influence the number of clinics required to meet the goal of vaccinating a majority of the population in a short time period. We recognized that the design and staffing of clinics may differ depending on certain characteristics, such as being a private or a public facility. Reaching out to various facilities in the private sector could be an option to fulfill the need for providing prophylaxis in a timely manner during a mass vaccination campaign. A possible optimistic assumption is that many clinicians, doctors, and nurses will volunteer to participate in the mass vaccination campaign if such an event occurs. Medical volunteers would certainly help reduce the burden of mass vaccination. Identifying and vaccinating potential volunteers, as well as providing specific training for a mass vaccination event, would be a crucial step to enhance the current medical surge capacity. To increase the chance that those organizations and individuals volunteer for mass vaccination, the Korean government may consider establishing a regulation providing protections from liability for potential adverse outcomes that occur as a result of responding to a medical surge. For instance, the USA has a regulation that authorizes the top health official to provide immunity from tort liability for those who respond to public health emergencies [20].

There is a concern that the genome of the smallpox virus is possible to build in a small lab with the necessary equipment [21]. This possibility was never considered during the smallpox eradication campaign that took place in the past. With enough motivation and resources, any rogue state or organization could try to build a virus and use it to leverage their political agenda. Given its disfiguring impact and high fatality rate, smallpox is something that mankind should always prepare for. Despite the very low likelihood of an outbreak, the detrimental impact of smallpox infection outweighs the burden of maintaining the smallpox vaccine stockpile, given the high efficacy of the vaccine. To justify and fulfill such a significant commitment, it is absolutely crucial to train medical personnel and responders properly and plan for successful implementation of mass prophylaxis.

## Figures and Tables

**Table 1.** Simulation inputs and parameters

Variables	Range
Input	
No. of core medical personnel	50-1,200 people
Per-clinic flow rate (patients/min/clinic)^1^	7.5-18.5
Duration of campaign (d)	10-1,080
No. of dispensing clinics	2-325
Simulation	
Population size	35,000,000 people
Hours of clinic operation per day	24 hr
Shifts per day	3 shifts
Downtime	15%
Crisis/isolation counseling	Included
Patient testing	Not included
Process time scenario	Fast process times
Event scenario	Large-scale event
Biological agen	Communicable disease (smallpox)

1 CDC recommendation is 10 patients/min/clinic.

**Table 2.** Number of dispensing clinics and per-clinic core staff based on per-clinic patient flow rate, when the campaign duration was fixed at 360 days

Flow rate (patients/min/clinic)	Dispensing clinic (A)	Core staff per clinic (B)	Total core staff (A×B)
7.5	10	100	1,000
8.5	8	114	912
9.5	8	127	1,016
10.5	7	140	980
11.5	6	154	924
12.5	6	167	1,002
13.5	6	181	1,086
14.5	5	194	970
15.5	5	207	1,035
16.5	5	221	1,105
17.5	4	234	936
18.5	4	247	988
